# Biological Abnormalities of Hemostasis in Patients with Epistaxis or Menorrhagia in Yaoundé, Cameroon

**DOI:** 10.1155/2024/6660891

**Published:** 2024-08-28

**Authors:** Annick Mintya Ndoumba, Aurélien Chendjou Kamela, Colince Wamba, Franklin Azebaze Agueguia, Nsa'Amang Eyebe Carolle, Claude Tayou Tagny, Dora Mbanya

**Affiliations:** ^1^ Faculty of Medicine and Biomedical Sciences University of Yaoundé I, Yaoundé, Cameroon; ^2^ Yaounde University Teaching Hospital, Yaoundé, Cameroon; ^3^ Higher Institute of Technology Applied to Health, Yaoundé, Cameroon; ^4^ Universite Libre de Bruxelles, Brussels, Belgium

## Abstract

**Introduction:**

In Cameroon, screening and diagnosis of minor hemorrhagic syndromes remain difficult and few research studies have been done to assess the magnitude of future bleeding risk and the burden of these disorders on quality of life. Epistaxis and menorrhagia are the two leading causes of bleeding disorders in the world population.

**Aim:**

The aim of this study was to investigate the biological abnormalities of hemostasis in patients with epistaxis and menorrhagia.

**Method:**

From January to December 2021, we conducted a cross-sectional study in six hospitals with a gynecology and ENT department. We selected patients who presented epistasis or menorrhagia through clinical file and made them pass an interview and biological exams. Venous blood collected on EDTA tube allowed us to measure full blood count, thin blood smear, and blood grouping. PT, APPT, and fibrinogen assay were measured from citrate platelet-poor plasma. This plasma stored at −20°C for a maximum of 3 months allowed us to measure vWF : Ag and vWF : CBA ELISA. The bleeding time was measured at the time of sampling.

**Result:**

In total, our study population consisted of 60 patients aged 01–45 years. Epistaxis (40%) and menorrhagia (29%) were the two main causes of bleeding complaints in our study, in addition to gingivorrhagia (15%) and prolonged bleeding after injury (03%). Almost 60% of the population had at least one abnormal hemostasis parameter. The main abnormalities found were low von Willebrand factor (30.19%), presence of macroplatelets (16.98%), prolonged bleeding time (15.09%), prolonged PT (15.09%), and low platelet count (¬07.55%).

**Conclusion:**

In Cameroon, bleeding disorders manifested by epistaxis and menorrhagia are mainly caused by abnormalities of primary hemostasis.

## 1. Introduction

Minor hemorrhagic syndromes (MHSs) are hemostasis disorders characterized by a “mild” or “moderate” tendency to bleeding and can have consequences on the quality of life of individuals [1]. In Cameroon, screening and diagnosis of MHS remains challenging and assessing the magnitude of future bleeding risk and the burden of these disorders on quality of life has been poorly studied and tends to be local practice based rather than guided by clinical, laboratory, or genetic criteria [2, 3]. The prevalence of MHS in patients with abnormal hemostasis varies according to published studies. This variability is partly related to the lack of a clear definition of minor bleeding disorders and the criteria used for diagnosis in the various studies [4, 5]. These disorders must indeed be distinctive enough to be considered a disease, but with less severity compared to major bleeding disorders; and in case of major disorders, by the absence of spontaneous episodes [6, 7].

A recently published study (2018) on the diagnosis of MHS showed that of 240 people reporting having bleeding disorders, only 8.8% actually presented with hemostasis abnormalities [8]. Epistaxis and menorrhagia were the main bleeding disorders reported with, respectively, 24% of all participants and 59% among female participants. Epistaxis is the most frequent clinical pathology in Ear, Nose, and Throat (ENT) practice (60% of adults). It accounts for 15% of manifestations in patients with hemorrhagic syndromes and is the 3rd symptom of rare bleeding disorders [9–11]. In the general population, menorrhagia represents 12% of specialized gynecological consultations. Also, 44% of women mention suffering from it and 5–20% of women aged between 30 and 49 consult for excessive blood loss [12, 13].

It is with a view to having a better knowledge of the causes of MHS in Cameroon that we proposed to study the biological abnormalities of hemostasis in patients presenting with epistaxis or menorrhagia and visiting the main hospitals in the city of Yaoundé. Specifically, it was to describe the sociodemographic characteristics of patients presenting with epistaxis or menorrhagia to identify the bleeding manifestations that accompany these bleeds and to determine the type and frequency of biological abnormalities of hemostasis in these patients.

## 2. Methodology

This is a cross-sectional, descriptive study that took place between January and December 2021 in six (06) hospitals in the city of Yaoundé with a gynecology department and an ENT department. These were the Yaoundé University Teaching Hospital, the Yaoundé Central Hospital, the Yaoundé General Hospital, the Yaoundé Gyneco-obstetrics and Pediatric Hospital, the Yaoundé Military Hospital, and the Essos Hospital Center. The biological tests were carried out at the hematology laboratory of the Yaoundé University Teaching Hospital. These are reference hospitals at the national level with a comprehensive care offer and a capacity of more than 300 hospital beds.

### 2.1. Participants

The study population consisted of any patient presenting with epistaxis or menorrhagia and seen as an outpatient in the gynecology or ENT departments in one of the listed hospitals. Those included in our study were patients who were medically followed in one of the hospitals, who had a complete clinical file, and who had given their informed consent to participate in the study. Those excluded from the study were patients with bad specimens and patients who had fever and who were under treatment that could influence the parameters of hemostasis.

### 2.2. Procedure

We regularly consulted the outpatient registers of the gynecology and ENT departments of health facilities in order to identify patients complaining of epistaxis and/or menorrhagia. We then selected those with a complete clinical file and contacted them by phone to involve them in the study. We went to meet the patients who agreed to participate in the study in order to interview through the standard questionnaire for the study and to do the biological samples. These involved blood samples from each participant at the bend of the elbow through the Vacutainer vacuum system successively using dry tubes, 3.2% sodium citrate anticoagulant tubes, and EDTA anticoagulant tubes. The samples were kept and transport in a portable insulated cooler at a temperature between 2 and 8°C for a maximum of 4 hours to be analyzed. The analyses performed with whole blood with EDTA anticoagulant tubes were full blood count, thin blood smear, and blood grouping. Platelet-poor citrated plasma was obtained after centrifugation at 2500 g for 15 minutes of venous blood sample mixed with the anticoagulant sodium citrate (concentration 0.109 M) at the rate of 9 parts of blood for 1 part of anticoagulant and was used for PT, APPT, and fibrinogen assay. The World Federation of Hemophilia (WFH) recommends the use of platelet-poor plasma in most coagulation tests in order to avoid any platelet interference in the in vitro analytical process [14]. Platelet-poor citrated plasma was then used for determination of VWF antigen (vWF : Ag) and measurement of VWF adhesive properties (vWF : CBA Collagen Type I). TECHNOZYM® vWF : Ag ELISA and TECHNOZYM® vWF : CBA ELISA Collagen Typ I are the reagents we used in this study, and its manufacturer (Technoclone) recommends storing the samples at −20°C for 3 months to guarantee the quality of analyses. In this study, we stored the samples at −20°C and analyzed them every 3 months in order to respect these recommendations while optimizing reagent consumption because for financial reasons we could not afford to do ELISA tests at a lower frequency. Note that the examination of the bleeding time (BT) was carried out immediately after the blood samples of each participant.

As part of this work, we did not use a control population because we initially have the reference values generated by the laboratory concerning the assayed parameters, to which we compared all our results.

The interpretation of the results was specific to each analysis technique used. Thus, a prolongation of the BT was defined by a value of the BT greater than 5 minutes; thrombocytopenia was defined by a platelet level below 150 G/L; von Willebrand factor deficiency was defined by a vWF : Ag value less than 0.5 U/ml (reduced level) for people with blood groups A, B, or AB and less than 0.35 U/ml for those with blood group O; von Willebrand factor deficiency was also defined by an isolated vWF : CBA value less than 0.6 U/ml (reduced level); PT prolongation was defined by a PT value less than 70%; aPTT prolongation was defined by a patient aPTT/control aPTT ratio greater than 1.2; and fibrinogen deficiency was defined as a fibrinogen level less than 200 mg/dl.

### 2.3. Analyze Method

The data for this study were collected using Microsoft Office Excel 2013 software and statistical analysis was performed using Epi Info 7 software. The graphs were created using Microsoft Office Excel 2013 software. Position parameters such as the mean and median, and dispersion parameters such as standard deviation and interquartile ranges, were used for the description of continuous variables. The categorical variables were described in terms of number and percentage.

This study received ethical clearance from the Institutional Research Ethics Committee of the Faculty of Medicine and Biomedical Sciences of the University of Yaoundé 1 (Ref.: 476/UY1/FMSB/VDRC/DAASR/CSD of February 14, 2020) and research authorisations from the different general managements of the hospitals where the study took place.

The biological data of the participants used to support the conclusions of this study have been deposited in the archives of the hematology and blood transfusion service of the Yaounde University Teaching Hospital.

## 3. Results

### 3.1. Circumstances of Bleeding Discovery

During the study period from January to December 2021, we recruited 60 participants. Epistaxis accounted for 70% (36 participants) of the circumstances of bleeding discovery compared to 30% for menorrhagia (24 female participants). The median age of onset of bleeding was 18 years and 75% of the population had an age of onset of less than 22 years (IQR = (13–22 years)).

### 3.2. Frequency and Intensity of Reported Hemorrhagic Complaints

**Figure 1:**
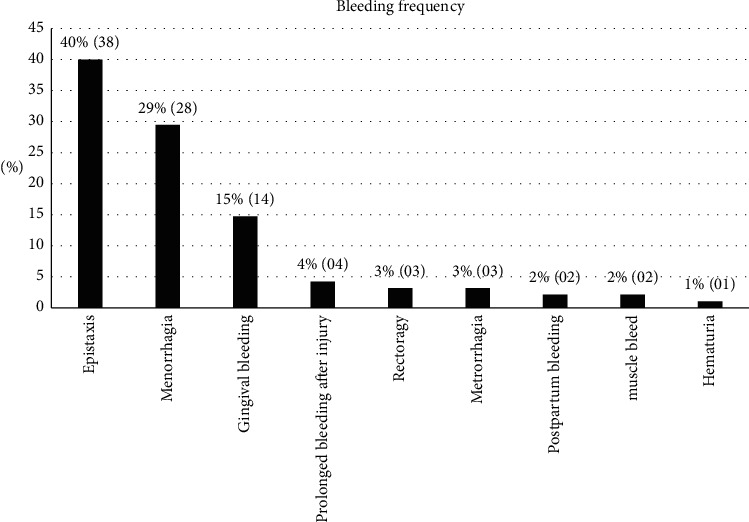
Frequencies of reported hemorrhagic complaints.

As shown in Figure 1, epistaxis (40%) and menorrhagia (29%) were the two main causes of bleeding complaints reported in our study, in addition to gingival bleeding (15%), prolonged bleeding after injuries (04%), and metrorrhagia and rectal bleeding (03%).

### 3.3. Frequency and Intensity of Bleeding

Eighty percent (48 participants) of the participants in our study reported moderate (31 participants) or major (17 participants) bleeding. Also, 48.33% of the participants had only one bleeding episode in the past six months, compared to 46.67% who had more than four episodes (Table 1).

### 3.4. Biological Abnormalities of Hemostasis

Thirty-four participants (34) out of 60 had at least one abnormal hemostasis parameter (56.67%) with a *p* value = 0.058. Primary hemostasis abnormalities accounted for 75.5% (37 abnormalities) of the abnormal parameters found (Table 2). Thus, the diseases likely to be responsible for these bleeding disorders are mainly von Willebrand disease type 1 (20.45%) or type 2 (10, 20%), functional platelet deficiency (26.53%), and hypovitaminosis *K* or factor VII deficiency (16.33%).

### 3.5. Sociodemographic Characteristics of the Study Population

The age of our study population was between 01 and 45 years old. The median age was 23.5 years old (IQR = (20–29.75 years)) and the most represented age group was (20–24). Students were the most represented profession (*n* = 25; 45%) and women were more numerous than men (sex ratio = 0.33).  Table 3 summarises the sociodemographic characteristics of our study population.

## 4. Discussion

During the study, we recruited 60 participants. Epistaxis (70%) and menorrhagia (30%) were the only causes behind the discovery of bleeding disorders in our population because they were the hemorrhagic manifestations retained as the selection criterion for participants. However, 41% of female participants mentioned menorrhagia as a circumstance of discovery of bleeding; Tosetto et al. found similar data (47%) in 2013 [15]. Similarly, although 75% of the participants had an age of discovery of bleeding less than 22 years, the median age was relatively high (18 years). Tossetto et al. explained that the risk of bleeding is much lower during the first years of life and that even by modeling an exponential increase in the risk of bleeding with age (assumption made to take into account the specific problems of women and traumatic bleeding or surgical procedures, leading to a higher risk of bleeding in adults than in infants [16]), the probability of having a minor bleeding symptom by the age of 30 can be as high as 33% [15].

Epistaxis (40%) and menorrhagia (29%) were the two main causes of hemorrhagic complaints reported during our study, in addition to gingival bleeding (15%), prolonged bleeding after injury (04%), and metrorrhagia and rectorrhagia (03%). With more or less varied frequencies, several authors have identified these hemorrhagic manifestations in similar studies [12, 17].

In addition, more than 80% of the participants declared as being “moderate” or “high” the intensity of the bleeding disorders from which they suffered. Even more, we found a marked difference between participants who experienced only one bleeding episode in the past six months (48.33%), those who experienced 2 or 3 (5%), and those who experienced more than four (46.67%). This can be explained by the fact that the lack of standard measurement criteria retained for the identification of bleeding episodes, it is possible that trivial (nonsignificant) bleeds that should not be taken into account have been added to bleeds true bleeding that may be representative of an underlying disease. Indeed, some patients often have a more subjective perception of hemorrhagic manifestations and generally declare them more often than they actually have [2, 6].

Nearly 60% of the patients had at least one abnormal hemostasis parameter, mainly parameters exploring primary hemostasis (75.5%). These results are rather consistent with the type of bleeding found (mainly mucosal bleeding). Indeed, these are distinctive enough to be considered a disease, but with less severity compared to major bleeding disorders which mainly affect the skin tissue and joints and are generally caused by abnormalities in plasma coagulation [2]. However, these data differ from those of Vries et al. in 2018 who found a proportion of 8.8% of the participants with at least one abnormal hemostasis parameter [8]. Mokhtar et al. had obtained a proportion of 23.3% [12]. The significant difference in proportion of our study can be explained by our recruitment method, which notably consisted in calling the participants through their phone number, whereas Mokhtar et al. and Vries et al. recruited participants in their respective studies at the time of the consultation. It could turn out that the majority of the members of our study had a real diagnostic interest for a health problem that had not found a solution and persisted over time.

Primary hemostasis abnormalities accounted for 75.5% (37 abnormalities) of the abnormal parameters found. Among other things, it was von Willebrand factor deficiency which was found in 16 participants (30.19%). Other primary hemostasis abnormalities were presence of macroplatelets (16.98%), prolonged bleeding time (15.09%), low platelet rate (07.55%), and platelet aggregates (07.55%). Similar results had been found in many studies although with different proportions [12, 17, 18]. Thus, Saxena et al. reported in 2003 that von Willebrand's disease is the most common inherited pathology of hemostasis found in women in India with menorrhagia (11.9%), as reported in the Caucasian population [18]. Mouktar et al. demonstrated that platelet abnormalities (72.7%), mainly thrombocytopenia (54.3%), are the main hemostasis abnormalities responsible for bleeding disorders during a study that identified 2,949 patients over 16 years of hematological consultations in a clinic in Egypt [12].

The population consisted of females, 45 participants (75%) compared to male, 15 participants (25%). Indeed, as reported by Vries et al. in 2018 [8], women are more likely to complain of a bleeding disorder (71%). In addition, Fontana and Boehlen reported in 2007 that 65% of women and 35% of men answered “yes” to the question “Do you have a bleeding problem?” [6]. As for the average age of our study population (IQR = (20–29.75 years)), it corresponds to that of young adults and corroborates with the professional category mainly found among our participants (students at nearly 50%).

## 5. Conclusion

This study is a first in Cameroon reporting biological abnormalities of hemostasis in patients with minor bleeding disorders. It appears that patients with epistasis or menorrhagia mainly present biological abnormalities related to primary hemostasis, the most common being von Willebrand's disease. This disorder, very little studied in Cameroon because of the difficulty of diagnosis, must, however, retain special attention because of the complications it can cause on the quality of life of individuals.

## 6. Study Limitations

Our study concerns a small size (60 participants) of the population studied. Studies on large populations should be carried out in order to verify the observations made within the framework of this work. Also, other biological parameters could be explored to have a better appreciation of hemostasis abnormalities in patients with epistaxis and menorrhagia. Specifically, platelet functional deficiency is one of the coagulation abnormalities most frequently found in our study and indeed deserves greater visibility and better exploration. Unfortunately, we did not carry out the additional tests necessary for its exploration within the framework of this study and could be the subject of a further relevant study.

## Figures and Tables

**Table 1:** Frequency and intensity of bleeding.

Bleeding intensity	*n*	Proportion (%)

Low	12	20.00
Moderate	31	51.67
High	17	28.33

Bleeding episodes over the past six months	*n*	Proportion (%)

1 time	29	48.33
2 times	3	5.00
3 times	0	0.00
4 or more times	28	46.67

**Table 2:** Interpretation of biological abnormalities of hemostasis.

Hemostasis abnormalities	Possible diseases	*n*	Proportion (%)
Primary hemostasis disorder	*von Willebrand disease 1* vWF: Ag lowered vWF: CBA normal or downgraded Normal or elongated APTT Normal or elongated BT	11	22.45
	*von Willebrand disease 2* vWF: CBA lowered vWF: Ag normal Normal or elongated APTT Normal or elongated BT	5	10.20
	*Thrombocytopenia* Platelet rate lowered Normal or elongated BT	4	8.16
	*Dic* Lowered PLT rate Normal or elongated BT Normal or elongated PT Normal or elongated APTT	4	8.16
	*Functional platelet deficiency*: Presence of macro platelets and/or platelet aggregates Normal or low platelet count Normal or elongated BT	13	26.53

Disorder of the extrinsic coagulation pathway	*Hypovitaminosis K, factor VII deficiency*: Elongated PT Normal or lowered APTT	8	16.33

Intrinsic coagulation pathway disorder	*Presence of antiphospholipid antibodies* Factor XII deficiency Factor XI deficiency Factor IX deficiency Or factor VIII deficiency Elongated APTT Normal or lowered PT	3	6.12

Others	*Fibrinogen deficiency* Lowered fibrinogen level Normal PT and APTT	1	2.04

**Table 3:** Sociodemographic characteristics of our study population.

Sociodemographic characteristics	Number (*n* = 60)	Percentage (%)
*Age*		
0–4 years	4	6.67
5–9 years	0	0.00
10–14 years	1	1.67
15–19 years	8	13.33
20–24 years	20	33.33
25–29 years	12	20.00
30–34 years	7	11.67
35–39 years	3	5.00
40–44 years	2	3.33
45–50 years	3	5.00

*Gender*		
Male	15	25.00
Female	45	75.00

*Profession*		
Child	3	5.00
Merchant/seller	9	15.00
Pupil	5	8.33
Teacher/professor	3	5.00
Student	27	45.00
Nurse/lab technician	3	5.00
Physician/biologist	8	13.33
Housewife	2	3.33

## Data Availability

The biological data of the participants used to support the conclusions of this study have been deposited in the archives of the hematology and blood transfusion service of the Yaounde University Teaching Hospital.
